# Deep Cryogenic Treatment Characteristics of a Deformation-Processed Cu-Ni-Co-Si Alloy

**DOI:** 10.3390/ma15093051

**Published:** 2022-04-22

**Authors:** Keming Liu, Xiaochun Sheng, Xiaolong Li, Mulin Li, Zhi Shen, Kai Fu, Haitao Zhou, Andrej Atrens

**Affiliations:** 1 Jiangxi Key Laboratory for Precision Actuation and Control, Nanchang Institute of Technology, Nanchang 330099, China; 2016984620@nit.edu.cn (X.S.); 15705182791@163.com (M.L.); 2009994122@nit.edu.cn (Z.S.); 2015994551@nit.edu.cn (K.F.); 2 Engineering Technology Research Center, Zhongye Changtian International Engineering Co., Ltd., Changsha 410205, China; l554490@163.com; 3 School of Materials Science and Engineering, Central South University, Changsha 410083, China; htzhou@csu.edu.cn; 4 Centre for Advanced Materials Processing and Manufacturing, The University of Queensland, Brisbane, QLD 4072, Australia; andrejs.atrens@uq.edu.au

**Keywords:** Cu alloy, cold rolling, DCT, microstructure, properties

## Abstract

This paper investigated the influence of deep cryogenic treatments (DCT) on the tensile strength, elongation to fracture and conductivity of a deformation-processed Cu-Ni-Co-Si alloy. The tensile properties were measured using a mechanical testing machine. The conductivity was evaluated using a low-resistance tester. The microstructure and precipitated phases were analyzed using scanning electron microscopy (SEM), transmission electron microscopy (TEM), an energy dispersive spectrometer (EDS) and an X-ray diffractometer (XRD). The tensile strength, elongation to fracture and conductivity of the Cu-1.34Ni-1.02Co-0.61Si alloy before and after cold rolling at 47% reduction increased with increasing DCT time and tended to be stable at about 36 h. The microstructure became more uniform after the DCT. The grain size was refined and was smallest after DCT for 48 h. The DCT promoted the precipitation of the solid solution elements Ni, Co and Si from the Cu matrix to form many fine and evenly distributed 20–70 nm spherical second-phase particles in the grains and grain boundaries.

## 1. Introduction

High-strength Cu-based alloys have been significant materials in many industrial fields over the past few decades [1,2,3,4,5,6]. Especially Cu-Ni-Si alloys have been widely used for lead frame materials due to their high strength, good conductivity and non-magnetic characteristics [7,8,9,10]. The C7035 alloy is one of the important Cu-Ni-Si alloys. Its main chemical composition is 1.0~2.5% Ni, 0.5~1.2% Si, 1.0~2.0% Co and balanced Cu. The C7035 alloy has service performance significantly better than that of the C7025 alloy due to the addition of Co [11,12,13,14]. The rapid development of the electronic industry has caused integrated circuits to have higher requirements for the strength and conductivity of Cu-Ni-Si alloys.

Liao et al. [15] prepared a Cu-Ni-Co-Si alloy by temperature-controlled mold continuous casting (TCMCC) and cold rolling. The strength and conductivity of the alloy increased by 327 MPa and 0.6% IACS after a cumulative cold rolling reduction of 97.5%. Suzuki et al. [16] found that the addition of trace iron, cold deformation and a suitable aging treatment increased the strength and ductility and proposed a novel multi-step aging treatment coupled with cold deformation to obtain a Cu-Ni-Si alloy with high strength and good conductivity. Huang et al. [17] produced a Cu-Ni-Co-Si alloy treated by a two-step thermo-mechanical process with tensile strength, conductivity and elongation of 1086 MPa, 30.1% IACS and 3.6%. Zhao et al. [18] fabricated a Cu-Ni-Co-Si alloy treated by a multi-stage thermo-mechanical treatment to have a tensile strength, conductivity and elongation of 810 MPa, 57.5% IACS and 3.4%. Ban et al. [19] designed and prepared a novel Cu-Ni-Co-Si alloy to have a microhardness, conductivity and elongation after aging treatment of 300 HV, 42.8% IACS and 7%.

DCT, also known as an ultra-low temperature treatment, refers to the experimental method of using low-temperature liquid substances such as liquid nitrogen as refrigerant to heat and treat materials below −130 °C for improving material properties. In the past decade, DCT has been an important method to increase the properties of ferrous and amorphous alloys as well as some non-ferrous alloys [20,21,22,23,24,25,26,27]. The factors affecting the effectiveness of DCT include DCT holding time, DCT holding temperature, the cooling/warming rate of DCT and the placement of DCT in the preparation scheme. Jovičević-Klug et al. [28] investigated the effect of DCT on the microstructure and microstructural evolution of stainless steel, cold-work tool steel, bearing steel and hot-work tool steel and found that the DCT increased the precipitation of carbides. The precipitates were more spherical, smaller and more uniformly distributed. The effectiveness of DCT on the matrix is mainly related to the homogenization of the matrix and the redistribution of alloying elements. Barylski et al. [29] studied the influence of DCT on the microstructure, wear and micromechanical properties of a Mg-Y-Nd-Zr alloy and found that the DCT accelerated the precipitation of solid solution atoms to increase the strength of the Mg-Y-Nd-Zr alloy, reduced micro-cutting and the formation of deep scratches in the abrasion process of the Mg-Y-Nd-Zr alloy, to increase the service life of the alloy. Su et al. [30] researched the impact of DCT on the corrosion resistance and strength of a high strength 7075 aluminum alloy. The DCT increased remarkably the corrosion resistance of the aluminum alloy without sacrificing the strength. This was due to the fact that more η’ phase was precipitated from the matrix, the grain boundary precipitates were discontinuous, and the precipitate-free zone was wider.

Previous investigations indicated that large-scale integrated circuits have higher requirements for the strength and conductivity of Cu-Ni-Si alloys. DCT is a promising method to increase these properties. Accordingly, this paper focused on the effect of DCT on the microstructure, tensile properties and conductivity of a Cu-Ni-Co-Si alloy. The aims were to optimize the thermo-mechanical treatment and to increase the strength, ductility and conductivity of the alloy, to make it meet the application needs of different high-tech fields.

## 2. Experimental Procedures

The experimental material is a commercial Cu-Ni-Co-Si alloy strip. The initial width and thickness were 28.00 mm and 0.30 mm. The chemical composition is shown in Table 1.

The Cu-Ni-Co-Si alloy strip was cold rolled at room temperature using a four-high mill. The roll diameter and width were 220 mm and 110 mm, respectively. The roll rotation speed was 19 r/min. The cold-rolled sample was produced by four pass cold rolling with a reduction of 0.05 mm, 0.04 mm, 0.03 mm and 0.02 mm, respectively. The sample thickness was 0.16 mm and the rolling reduction was 47%. The cold-rolled samples were placed in a sample holder. The sample holder was immersed in a liquid nitrogen tank for the DCT. The DCT times were 0 h, 6 h, 12 h, 24 h, 36 h and 48 h, respectively. The sample holder was taken out and warmed in air to room temperature.

**Figure 1:**
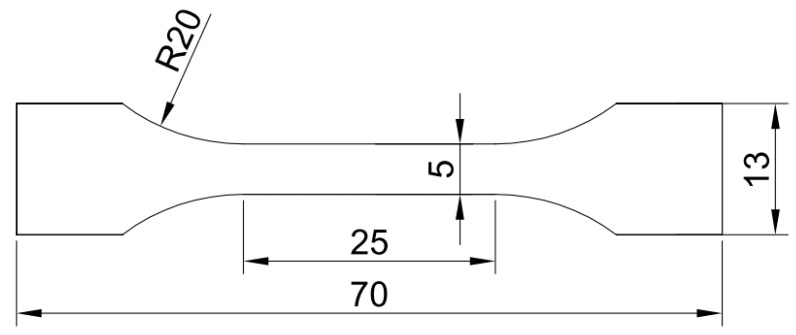
Geometry of samples for a tensile test.

The samples for microstructure observation were prepared by standard metallographic sample preparation method and were etched using a solution of 5 g FeCl_3_, 25 mL HCl and 100 mL H_2_O. The DCT microstructure of the alloy strip was characterized using a scanning electronic microscope (SEM). The precipitates of the DCT alloy were characterized using a transmission electron microscope (TEM). The precipitated phase of the aged alloy was analyzed using a X-ray diffractometer (XRD) and an energy dispersive spectrometer (EDS) installed in the TEM. The tensile properties of the alloy samples were measured using an mechanical testing machine. Figure 1 presents the geometry of samples for a tensile test. The orientation was parallel to the cold rolling direction. The conductivity of the alloy samples was evaluated using a DC low-resistance tester. Each sample was measured at least 3 times, and the average value was taken as the test result.

## 3. Results

### 3.1. Tensile Strength

**Figure 2:**
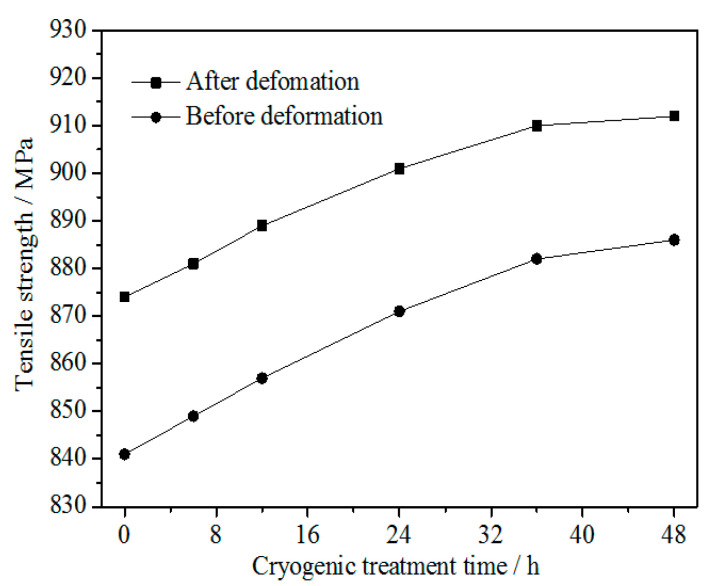
Tensile strength of the Cu-1.34Ni-1.02Co-0.61Si alloy before and after cold rolling at 47% reduction with the DCT time in liquid nitrogen.

Figure 2 shows the tensile strength of the Cu-1.34Ni-1.02Co-0.61Si alloy before and after cold rolling at 47% reduction with the DCT time in liquid nitrogen for 0 h, 6 h, 12 h, 24 h, 36 h and 48 h. Figure 2 indicates that, similar to the results of previous investigations [2,15,24,31], (i) the tensile strength of the Cu-1.34Ni-1.02Co-0.61Si alloy after cold rolling at 47% reduction was significantly higher than that before cold rolling, which was attributed to work hardening and fine grain strengthening, and (ii) the tensile strength of the Cu-1.34Ni-1.02Co-0.61Si alloy after cold rolling with DCT was higher than before cold rolling at each holding time. In addition, the tensile strength of the alloy before and after cold rolling at 47% reduction increased with increasing DCT time and tended to be stable after ~36 h, as shown in Figure 2. During the initial stage of DCT, the tensile strength increased quickly with DCT time. With increasing treatment time, the growth rate of the tensile strength gradually slowed down. After the DCT time of 36 h, the tensile strength essentially remained constant and unchanged with a further increase in time.

### 3.2. Elongation

**Figure 3:**
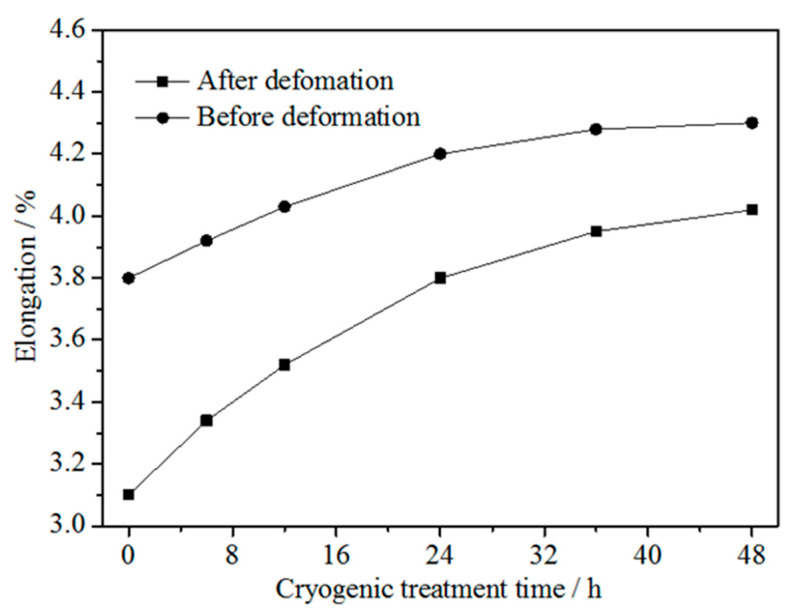
Elongation to fracture of the Cu-1.34Ni-1.02Co-0.61Si alloy before and after cold rolling at 47% reduction with the DCT time in liquid nitrogen.

Figure 3 shows the elongation to fracture of the Cu-1.34Ni-1.02Co-0.61Si alloy before and after cold rolling at 47% reduction and with the DCT time in liquid nitrogen for 0 h, 6 h, 12 h, 24 h, 36 h and 48 h. Similar to the effect of DCT on tensile strength, the elongation to fracture of the alloy before and after cold rolling at 47% reduction increased with increasing DCT time and tended to be stable after ~36 h. During the initial stage of DCT, the elongation to fracture increased rapidly with DCT time. With increasing treatment time, the growth rate of the elongation to fracture gradually decreased. After the DCT time of 36 h, the elongation to fracture of the alloy was essentially constant and unchanged with a further increase in time. In addition, the elongation to fracture of the Cu-1.34Ni-1.02Co-0.61Si alloy after cold rolling at 47% reduction was lower than that before cold rolling at each treatment time. However, the difference of elongation to fracture between the undeformed and cold-rolled alloy decreased with increasing DCT time, as shown in Figure 3.

### 3.3. Conductivity

**Figure 4:**
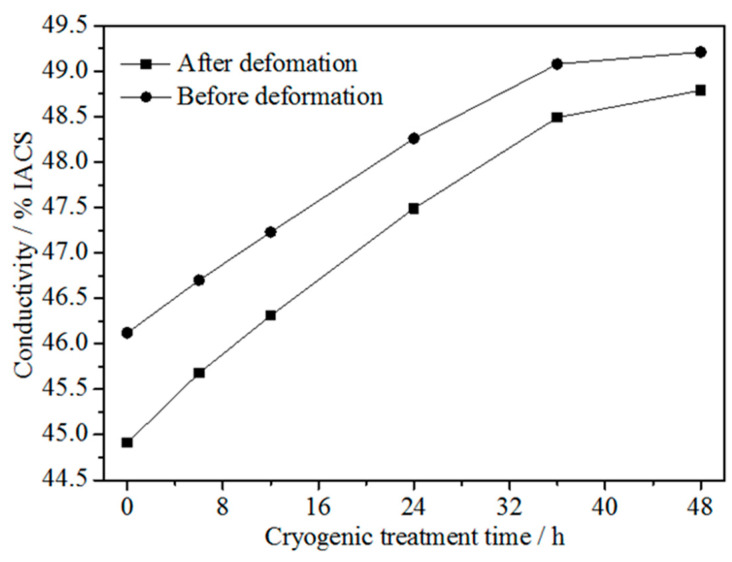
Conductivity of the Cu-1.34Ni-1.02Co-0.61Si alloy before and after cold rolling at 47% reduction with the DCT time in liquid nitrogen.

Figure 4 shows the conductivity of the Cu-1.34Ni-1.02Co-0.61Si alloy before and after cold rolling at 47% reduction with the DCT time in liquid nitrogen for 0 h, 6 h, 12 h, 24 h, 36 h and 48 h. Figure 4 presents that the conductivity of the alloy before and after cold rolling at 47% reduction increased with increasing DCT time, and tended to be stable after ~36 h. During the initial stage of DCT, the conductivity increased rapidly with increasing DCT time. Thereafter, the growth rate of conductivity with increasing DCT time gradually slowed down. After the DCT time of 36 h, the conductivity of the alloy was essentially constant and unchanged with a further increase in time. Similar to the results of previous investigations [16,19,32], the conductivity of the Cu-1.34Ni-1.02Co-0.61Si alloy after cold rolling at 47% reduction was lower than that before cold rolling at each treatment time. However, the difference of conductivity between the undeformed and cold-rolled alloy decreased with increasing DCT time, as shown in Figure 4.

## 4. Discussion

### 4.1. Microstructure

**Figure 5:**
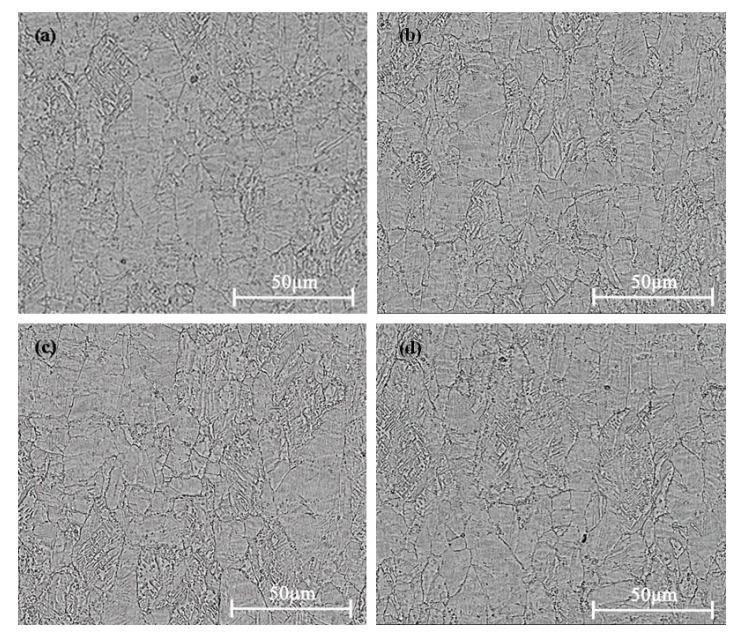
Microstructure of the Cu-1.34Ni-1.02Co-0.61Si alloy after cold rolling at 47% reduction with the DCT time in liquid nitrogen for (**a**) 0 h, (**b**) 6 h, (**c**) 24 h and (**d**) 48 h.

Figure 5a shows the microstructure of the Cu-1.34Ni-1.02Co-0.61Si alloy after cold rolling at 47% reduction. Figure 5b–d present the effect of DCT on the microstructure of the Cu-1.34Ni-1.02Co-0.61Si alloy after cold rolling at 47% reduction. Figure 5 shows that the grain size of the Cu-1.34Ni-1.02Co-0.61Si alloy was refined, and the microstructure became more uniform after the DCT. The average grain size of the alloy with the DCT for 48 h was the smallest. Image Pro Plus calculation showed that the average grain size of the Cu-1.34Ni-1.02Co-0.61Si alloy without DCT was about 35 µm, and that with the DCT for 48 h was about 30 µm. The DCT caused the sharp temperature reduction to decrease the internal grain gap and to shrink the volume of the alloy, which reduced the internal defects of the alloy. In addition, similar to the principle of thermal expansion and cold contraction, the reduction in volume caused compressive stress in the alloy, which decreased the concentration of defects such as vacancies and increased the density of the alloy. At the same time, fine dispersed second-phase particles precipitated in the alloy with increasing DCT time, as shown in Figure 5d. Accordingly, the tensile strength of the alloy before and after cold rolling at 47% reduction increased with increasing DCT time and tended to be stable after reaching ~36 h.

### 4.2. Fracture

**Figure 6:**
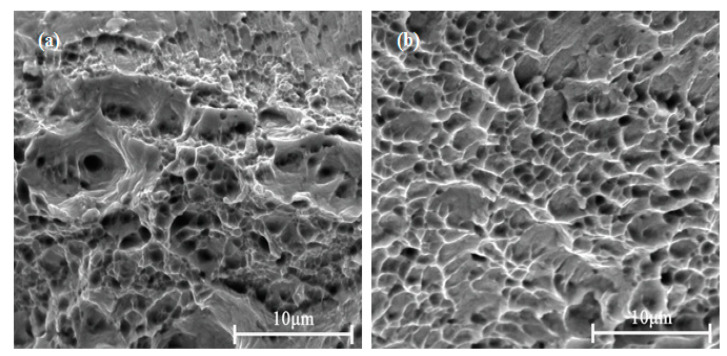
Fracture surface SEM images of the Cu-1.34Ni-1.02Co-0.61Si alloy after cold rolling at 47% reduction: (**a**) without DCT; (**b**) with the DCT in liquid nitrogen for 48 h.

Figure 6 presents the fracture surface SEM images of the Cu-1.34Ni-1.02Co-0.61Si alloy after cold rolling at 47% reduction without DCT and with the DCT in liquid nitrogen for 48 h. Both surfaces indicated ductile fracture. However, the dimple quantity of the alloy with DCT 48 h was larger, and the dimple distribution was more uniform than those without DCT. This is consistent with the smaller grains and more uniform distribution of grains in the alloy with DCT as shown in Figure 5. Those observations further suggested that the plasticity of the alloy could be increased by appropriate DCTs.

### 4.3. Precipitation

**Figure 7:**
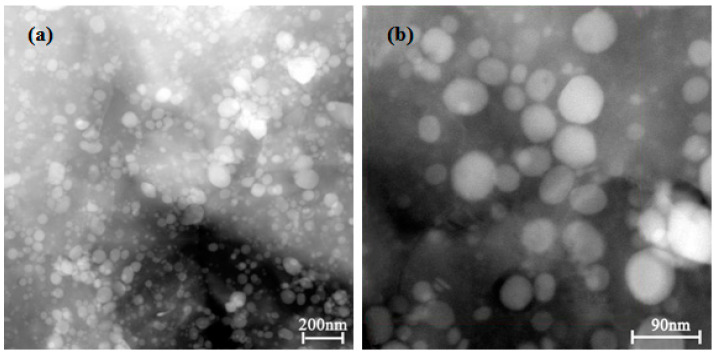
TEM images of the Cu-1.34Ni-1.02Co-0.61Si alloy after cold rolling at 47% reduction with the DCT in liquid nitrogen for 48 h: (**a**) microstructure of the matrix; (**b**) morphology and distribution of second phase.

Figure 7 presents the TEM images of the Cu-1.34Ni-1.02Co-0.61Si alloy after cold rolling at 47% reduction with the DCT in liquid nitrogen for 48 h. Consistent with the results observed by SEM microstructure, many fine and evenly distributed second-phase particles precipitated in the grain and grain boundary after the long time DCT, as shown in Figure 7a. The precipitated second-phase particles were basically spherical, and the size was between 20–70 nm, as shown in Figure 7b. This is because the DCT decreased rapidly the matrix temperature of Cu-1.34Ni-1.02Co-0.61Si alloy, which resulted in the sharp reduction in the solid solubility of Ni, Co and Si in the Cu matrix and caused the decline in the stability of the solid solution and the precipitation of Ni, Co and Si from the Cu matrix. At the same time, driven by the chemical potential, the precipitated atoms continued to diffuse and aggregate to form relatively stable fine dispersed second phases.

**Figure 8:**
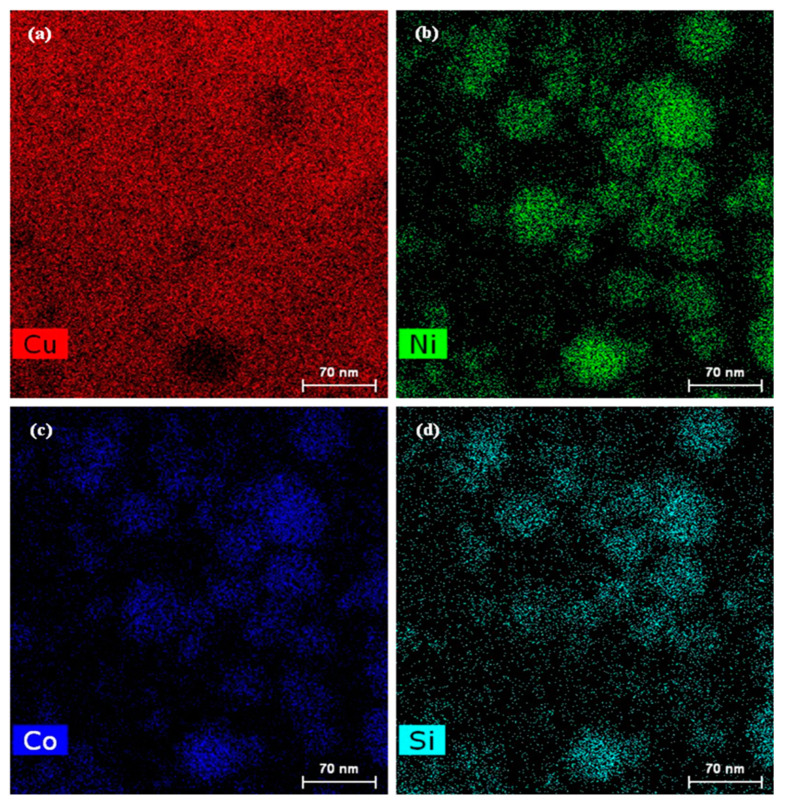
Area scanning images of the element distribution for the HADDF Z-contrast image of Figure 7b: (**a**) Cu; (**b**) Ni; (**c**) Co; (**d**) Si.

Figure 8 presents the area scan images of the element distribution for the HADDF Z-contrast image of Figure 7b. Figure 8a shows that the Cu was evenly distributed in the matrix, but the Cu content in some second-phase areas was relatively low, as shown by the black areas. There was little or almost no Cu in this kind of second phase. The contents of Ni, Co and Si in the Cu matrix were low; most of the Ni, Co and Si had precipitated from the Cu matrix and formed second-phase particles, but a small amount of these elements was still distributed in the alloy matrix, as shown in Figure 8b–d. In addition, Ni, Co and Si were not gathered in a single region, but in different spherical second-phase particles in the selected area, they corresponded to the aggregation of two or more kinds of elements, respectively. There were obvious different kinds of second phases, such as Cu-containing phases vs. Cu-free phases or Si-rich phases vs. Si-poor phases, as shown in Figure 8. The main elements of some second phases were Ni and Si and contained a very small amount of Cu and Co. The main elements of some second phases were Co and Si and contained a small amount of Cu and Ni. The main elements of other second phases were Cu, Ni, Co and Si, and the content of Si was high.

**Figure 9:**
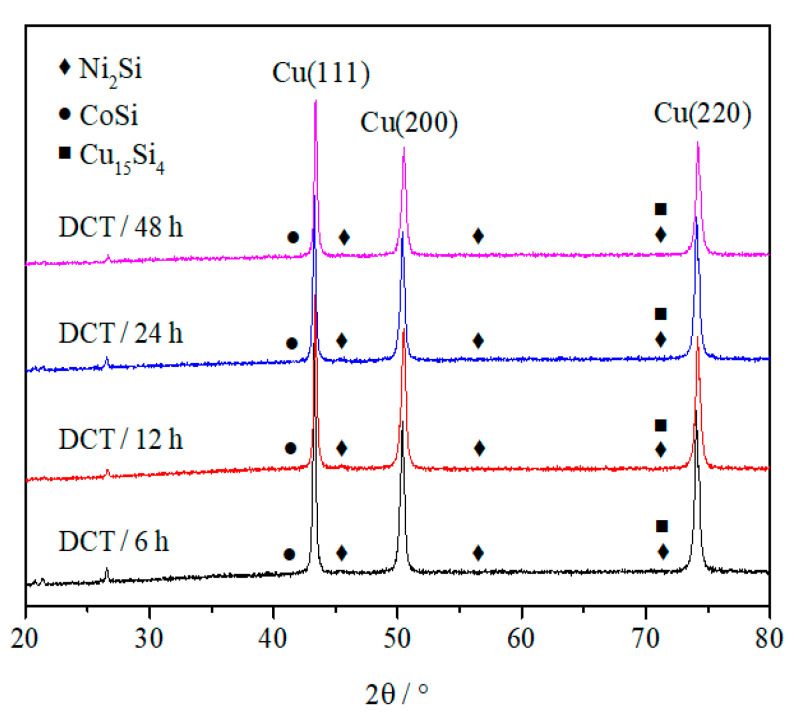
X-ray diffraction analysis of the Cu-1.34Ni-1.02Co-0.61Si alloy after cold rolling at 47% reduction with the DCT in liquid nitrogen for 6 h, 12 h, 24 h and 48 h.

Figure 9 shows the X-ray diffraction analysis of the Cu-1.34Ni-1.02Co-0.61Si alloy after cold rolling at 47% reduction with the DCT in liquid nitrogen for 6 h, 12 h, 24 h and 48 h. Figure 9 presents that the precipitated second-phase grains at all DCT times were Ni_2_Si, CoSi and Cu_15_Si_4_.

**Figure 10:**
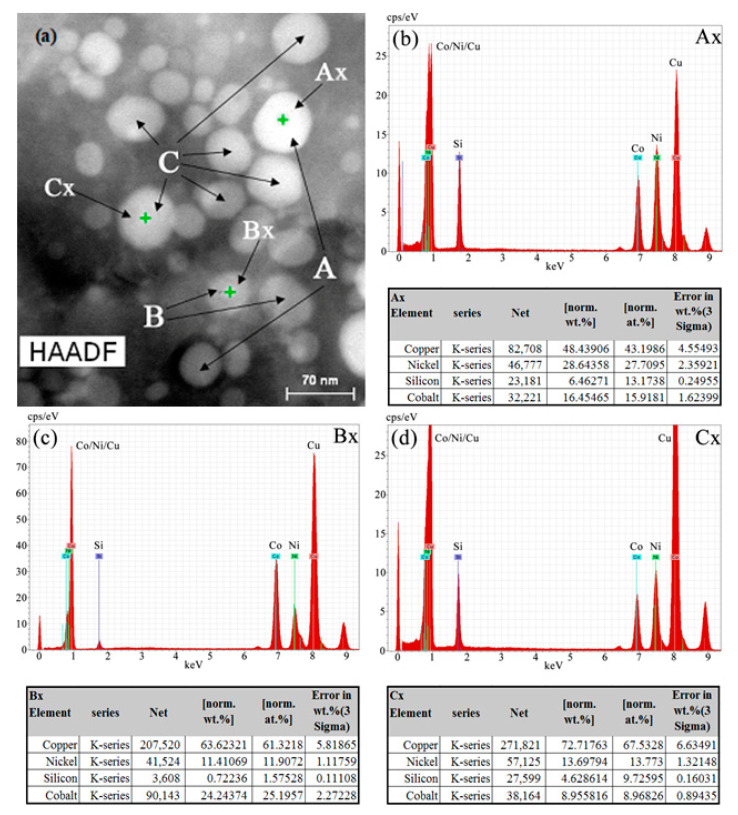
Component analysis of the second phase for the HADDF Z-contrast image of Figure 7b: (**a**) morphology of second phase, (**b**) energy spectrum of Ax, (**c**) energy spectrum of Bx, (**d**) energy spectrum of Cx.

Figure 10 presents the component analysis of the second phase for the HADDF Z-contrast image of Figure 7b. According to the area scanning images of the element distribution of Figure 8 and Figure 10a, the precipitated second phase in the DCT alloy could be divided into a Cu-containing phase, a Cu-free phase, a Si-rich phase and a Si-poor phase. Based on the area scanning results of element distribution, most of the precipitated fine dispersed phases were spherical, mainly including three types, as shown in Figure 10a. The main elements of the lightest second phase A were Ni, Co, Si and a very small amount of Cu, according to the area scanning images of the element distribution in Figure 8. The main elements of the darkest second phase B were Cu, Ni, Co and a very small amount of Si. The main elements of the darker second phase C were Cu, Ni, Co and Si, and the content of Si element was higher than that of the second phase B, as shown in Figure 8. Figure 10b–d show the EDS analysis of three representative second phases: Ax, Bx and Cx. Figure 10b,d present that the atomic ratio of Ni and Si in Ax was close to 2:1, and the atomic ratio of Co and Si in Cx was close to 1:1. This indicated that the precipitated second phase A in the alloy after aging treatment was Ni_2_Si. The precipitated second phase C was CoSi, according to the analysis result of X-ray diffraction as shown in Figure 9. The energy spectrum percentage of Bx showed that the atomic percentage of Si element was very low. Consequently, and making use of Figure 9, B was determined to be the Cu_15_Si_4_ phase.

The DCT promoted the fine and evenly distributed second-phase precipitates in the grains and grain boundaries. The number of precipitate particles increased with increasing DCT time. After reaching a certain time, the precipitation was basically complete. This caused the tensile strength of the alloy before and after cold rolling at 47% reduction to increase with increasing DCT time and to tend to be stable after a certain time. In addition, previous investigations [33,34,35,36] indicated that the conductivity of Cu alloys was not only influenced by the phonon, dislocation and interface scattering but also greatly affected by the internal impurities and defects. According to Matthissen rule, the resistivity of the Cu matrix *ρ*_Cu_ was mainly composed of four parts as follows [35]:

(1) $$\rho_{\mathrm{Cu}}=\rho_{\mathrm{pho}}+\rho_{\mathrm{dis}}+\rho_{\mathrm{int}}+\rho_{\mathrm{imp}}$$

where *ρ*_pho_, *ρ*_dis_ and *ρ*_int_ are the phonon scattering, dislocation scattering and interface scattering resistivity, respectively, and *ρ*_imp_ is the impurity scattering resistivity. Cu alloys with the same cold rolling reduction have similar *ρ*_pho_ and *ρ*_dis_. The interface between the Cu matrix and the second phase is similar for the Cu alloys with the same cold rolling reduction, so *ρ*_int_ is similar. The main differences in the evaluated conductivity were caused by the fine and evenly distributed second-phase precipitate particles. Accordingly, the conductivity of the alloy before and after cold rolling at 47% reduction increased with increasing DCT time and tended to be stable after reaching a certain time.

## 5. Conclusions

(1) The tensile strength of the alloy before and after cold rolling at 47% reduction increased with increasing DCT time and tended to be stable at about 36 h. These were due to the fact that the DCT promoted the fine and evenly distributed second-phase precipitates, decreased the concentration of defects such as vacancies and increased the density of the alloy, and these changes tended to be stable after a certain time. The tensile strength of the alloy after cold rolling at 47% reduction was higher than that before cold rolling.
(2) The elongation to fracture of the alloy before and after cold rolling at 47% reduction increased with increasing DCT time and tended to be stable at about 36 h. The dimple quantity of the alloy with DCT was larger than without DCT, and the distribution was more uniform.
(3) The conductivity of the alloy before and after cold rolling at 47% reduction increased with increasing DCT time and tended to be stable at about 36 h. These were due to the fact that the DCT promoted the precipitation of the solid solution elements Ni, Co and Si from the Cu matrix, and the precipitation and solid solution tended to balance after a certain time. The conductivity of the alloy before cold rolling was higher than that after cold rolling. However, the difference in conductivity decreased considerably with increasing DCT time.
(4) The grain size of the Cu-1.34Ni-1.02Co-0.61Si alloy was refined, the microstructure distribution became more uniform after the DCT, and the average grain size of the alloy with the DCT for 48 h was the smallest.
(5) The DCT promoted the precipitation of the solid solution elements Ni, Co and Si from the Cu matrix to form many fine and evenly distributed 20–70 nm spherical second-phase particles in the grain and grain boundary. The XRD and EDS analysis results indicated that the precipitated second-phase particles at all DCT times were Ni_2_Si, CoSi and Cu_15_Si_4_.
(6) In the future, DCT will be widely used in the field of high-strength high-conductivity copper alloys because DCT plays an important role in increasing the comprehensive properties of these alloys, which is of great significance to broaden the application range of copper alloys in high-tech fields.

## Figures and Tables

**Table 1:** Main chemical composition of a Cu-Ni-Co-Si alloy (wt.%).

Component	Ni	Co	Si	Cu
Content	1.34	1.02	0.61	Balance
